# Inference of dynamical gene-regulatory networks based on time-resolved multi-stimuli multi-experiment data applying NetGenerator V2.0

**DOI:** 10.1186/1752-0509-7-1

**Published:** 2013-01-02

**Authors:** Michael Weber, Sebastian G Henkel, Sebastian Vlaic, Reinhard Guthke, Everardus J van Zoelen, Dominik Driesch

**Affiliations:** 1, Leibniz Institute for Natural Product Research and Infection Biology – Hans Knöll Institute, Beutenbergstr. 11a, 07745 Jena, Germany; 2, BioControl Jena GmbH, Wildenbruchstr. 15, 07745 Jena, Germany, www.biocontrol-jena.com; 3 Department of Cell and Applied Biology, Radboud University, Heijendaalseweg 135, 6525 AJ Nijmegen, The Netherlands

**Keywords:** Gene-regulatory networks, Network inference, Heuristic algorithm, ODE, NetGenerator

## Abstract

**Background:**

Inference of gene-regulatory networks (GRNs) is important for understanding behaviour and potential treatment of biological systems. Knowledge about GRNs gained from transcriptome analysis can be increased by multiple experiments and/or multiple stimuli. Since GRNs are complex and dynamical, appropriate methods and algorithms are needed for constructing models describing these dynamics. Algorithms based on heuristic approaches reduce the effort in parameter identification and computation time.

**Results:**

The NetGenerator V2.0 algorithm, a heuristic for network inference, is proposed and described. It automatically generates a system of differential equations modelling structure and dynamics of the network based on time-resolved gene expression data. In contrast to a previous version, the inference considers multi-stimuli multi-experiment data and contains different methods for integrating prior knowledge. The resulting significant changes in the algorithmic procedures are explained in detail. NetGenerator is applied to relevant benchmark examples evaluating the inference for data from experiments with different stimuli. Also, the underlying GRN of chondrogenic differentiation, a real-world multi-stimulus problem, is inferred and analysed.

**Conclusions:**

NetGenerator is able to determine the structure and parameters of GRNs and their dynamics. The new features of the algorithm extend the range of possible experimental set-ups, results and biological interpretations. Based upon benchmarks, the algorithm provides good results in terms of specificity, sensitivity, efficiency and model fit.

## Background

For the adaptation of biological systems towards external and environmental stimuli usually a complex interaction network of intracellular biochemical components is triggered. That includes changes in the gene expression at both the mRNA and protein level. Considering a certain stimulus as an input signal to the system and mRNA or protein levels as outputs, the connecting network may include interactions between signal transduction intermediates: transcription factors and target genes. Generally, the term “gene-regulatory network” (GRN) summarises genetic dependencies, which describe the influence of gene expression by transcriptional regulation, [1].

The inference (elucidation) of GRNs is important for understanding intracellular processes and for potential manipulation of the system either by specific gene mutations, knock-downs or by treatment of the cells with drugs, e.g. for medical purposes. Towards a full understanding in terms of a complete network, partial models of the network give intermediate results which help to refine the knowledge and to design new experiments. Still, many gene-regulated cellular functions, e.g. stem cell differentiation, depend on more than one stimulus and the cross-talk within the GRN, e.g. [2]. On the other hand, the stimuli might influence distinct components of a GRN. Such biologically relevant dependencies can be investigated by applying two or more stimuli and measuring the influenced genes. This approach can be called multi-stimuli experiment. If this is carried out in two or more separate experiments, one derives multi-stimuli multi-experiment data. Only algorithms with the ability to consider those data can infer such dependencies.

As shown in review articles, e.g. [1,3,4], there are different inference methods using various sources of information thus leading to different results. Amongst the typically mathematical models the application of differential equations describing time-resolved gene expression data (“time series”) has been proven successful. Unfortunately the potential complexity of the networks leads to a high number of structural connections and parameters in contrast to the comparably small number of available measurement data. Apart from the problem of identifiability, the number of possible parameter combinations is very large, thus resulting in high computational costs. Therefore, appropriate heuristic approaches can reduce this amount while providing comparably good inference results. NetGenerator is a heuristic algorithm, which considers time series data to automatically infer GRNs influenced by an external stimulus, [5] and [6]. The approach combines a structure (network topology) and parameter optimisation. The final result in form of a differential equations model can be simulated and displayed graphically. An earlier version with less functionality was applied successfully to biological problems, e.g. the regulatory network of iron acquisition in *Candida albicans* and the analysis of the *Aspergillus fumigatus* infection process, [7] and [8].

In the present article, we propose NetGenerator V2.0, an extended version of the algorithm which enables the use of multi-stimuli multi-experiment data, thus increasing the number of addressable biological questions. This causes significant changes in the algorithmic procedures, especially the processing of this kind of data as well as the structure and parameter optimisation. Also, some other updated features will be outlined, for example the different modes of prior knowledge integration, further knowledge-based procedures, options of graphical outputs, changed non-linear modelling and re-implementation in the programming language / statistical computing environment R, [9]. Further, in comparison to the previous version, some of the algorithmic procedures will be explained in more detail, because they are important for understanding the overall method.

The successful application of the novel NetGenerator will be shown by inference of relevant multi-stimuli multi-experiment benchmark examples, namely systems with a different degree of cross-talk. Two aspects will be assessed: (i) reproduction of the benchmark systems (data and structure) and (ii) refinement / extension of a network structure by combination of different experimental data. Furthermore, the applicability of NetGenerator to a real-world problem is presented: after describing necessary data pre-processing steps, the underlying GRN of chondrogenic differentiation of human mesenchymal stem cells, a process influenced by the two stimuli TGF-beta1 and BMP2, is inferred.

## Methods

In the following subsections the necessary background knowledge and methodology of the NetGenerator algorithm is described. In comparison to previous publications this includes new, updated and more detailed algorithmic procedures. First, the motivation and the goals are defined by considering the biological data. Necessary steps of data pre-processing are also explained within this subsection. Subsequently, ordinary differential equations and some of their properties are presented as a means for modelling the dynamics of gene regulatory networks. Then the heuristic approach of the algorithm is explained including the structure and parameter identification (here: optimisation-based determination). The next important topic will be the consideration of prior knowledge, followed by a subsection about the numerical simulation as well as the representation of modelling and graphical results. Finally, some important options and their influence to the algorithm are presented.

### Time series data and pre-processing

**Figure 1:**
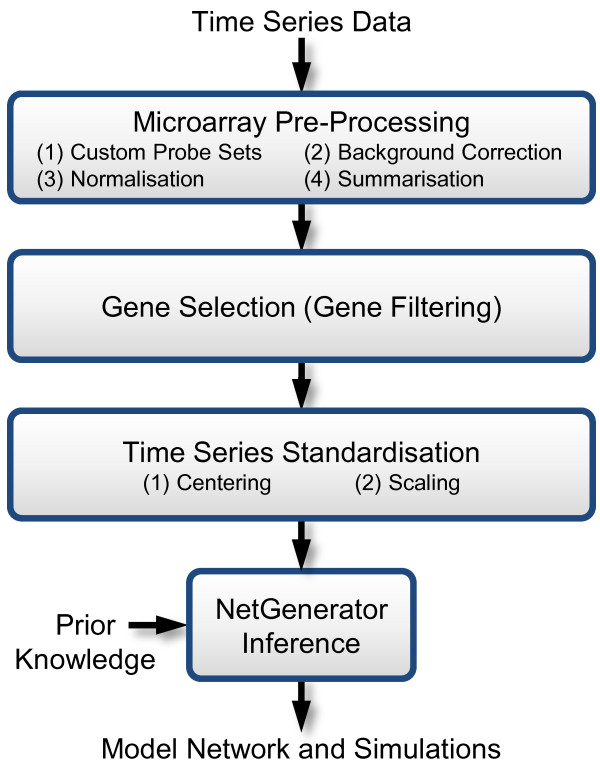
**Data pre-processing work flow.** This work flow illustrates inputs and outputs of NetGenerator as well as recommended data pre-processing steps: pre-processing of microarray data, selection of genes, standardisation of gene expression time series.

Gene expression time series data as required by NetGenerator are typically derived from microarray measurements. Before starting the network inference, raw microarray data have to be processed comprising a series of steps. The three main steps are displayed in Figure 1: (i) microarray pre-processing, (ii) gene selection and (iii) time series scaling.

Microarray pre-processing applies multiple procedures to remove non-biological noise from the data and to estimate gene expression levels. Custom probe-sets, as assembled by [10], reduce the number of cross-hybridising probes. This initial reduction accomplishes a one-to-one correspondence between probe-set and gene. Background correction, normalisation and summarisation are provided by the RMA package, [11], resulting in logarithmised gene expression estimates, which can be used for the next processing step.

Gene selection (“filtering”) is the important second step of processing, since reliable network inference is only feasible for a sufficient number of measurements per gene [1]. This number is often limited and therefore a selection of genes for modelling is inevitable. Candidate genes should show pronounced temporal dynamics and significant differences compared to the control group. Statistical methods for identification of differentially expressed genes are widely used for gene selection. We use the LIMMA tool, which can operate on time series data determining significance of gene expression changes over time [12]. The statistical test (moderated *t*-statistics) operates on contrast terms, defined by subtracting the control group at each time point. LIMMA returns a ranked table for all genes containing columns for gene name, fold-change and adjusted *p*-values. Differentially expressed genes are selected by a combination of adjusted *p*-value cut-off and fold-change criterion.

Time series standardisation is the last processing step including centering and scaling of each time series. The centering procedure subtracts the original initial value at the starting time point from all values such that the transformed time series starts from zero. In the subsequent scaling procedure each time series is divided by its maximum (absolute value) across all provided experimental data. This leads to gene-wise scaled data and gene expression time series varying within −1and 1. The resulting data provided to the NetGenerator algorithm are stored in ${\underset{=}{X}}_{e}$ and ${\underset{=}{U}}_{e}$, i.e. matrices for the time series (output) and stimuli (input) data, respectively, for all experiments *e* = 1,…,*E*. Therefore, the dimensions are ${\underset{=}{X}}_{e}\;:\;T_{e}\times N$ and ${\underset{=}{U}}_{e}\;:\;T_{e}\times M$ with *T*_*e*_ being the number of experimental time points, *N* being the number of time series and *M* being the number of inputs. Furthermore, NetGenerator provides the option of introducing additional artificial data points by cubic spline interpolation.

### GRNs considered as linear time-invariant systems

The NetGenerator algorithm infers dynamical models of GRNs. Their general non-linear dynamics can be described by a set of first-order time-invariant ordinary differential equations (ODEs), initial conditions, and time range (validity period)

(1) $$\begin{array}{ccc} \;\underset{-}{\dot{x}}(t) & \;= & \underset{-}{f}\left(\underset{-}{x}(t),\underset{-}{u}(t),\underset{-}{\theta }\right)\\ \;{\underset{-}{x}}_{0} & \;= & \underset{-}{x}(t_{0})\\ \;t & \;\geq & t_{0} \end{array}$$

with the vector of state variables $\underset{-}{x}$ and their changes $\underset{-}{\dot{x}}$ as a function $\underset{-}{f}$ of state variables, input vector $\underset{-}{u}$ and parameter vector $\underset{-}{\theta }$. The state variables and inputs depend on time *t*, the independent variable, that is dropped in further equations. The description is valid for a certain time range starting at *t*_0_ from the initial conditions for the state variables ${\underset{-}{x}}_{0}$. If not stated otherwise each of the state variables corresponds to one specific output variable, i.e. one time series. The dimensions of the variables are $\underset{-}{x}\;:\;N\times 1$, $\underset{-}{u}\;:\;M\times 1$, and $\underset{-}{\theta }\;:\;P\times 1$, with *N* being the number of state variables, *M* the number of inputs and *P* the number of parameters.

Even though NetGenerator has a non-linear modelling option, the core mechanisms are based on linear modelling. Under the assumption that most networks can be considered linear and time-invariant, the differential equation system in (1) can be modified resulting in the linear state-space equation system

(2) $$\underset{-}{\dot{x}}=\underset{=}{A}\;\underset{-}{x}+\underset{=}{B}\;\underset{-}{u}$$

with the system or interaction matrix $\underset{=}{A}\;:\;N\times N$ and the input matrix $\underset{=}{B}\;:\;N\times M$. Most important for the understanding of the biological systems properties and the heuristic approach of the NetGenerator algorithm is the system matrix $\underset{=}{A}$ and its elements *a*_*i*,*j*_, *i*,*j* ∈ *N*, because they describe the dynamics and the coupling of state variables.

Under the assumption, that the behaviour of a GRN is described sufficiently by indirect transcriptional events and not by a conversion of material, activation (*a*_*i*,*j*_ > 0) or inhibition (*a*_*i*,*j*_ < 0) of state variable *x*_*i*_ is not changing the value of the originating state variable *x*_*j*_.

Without any further assumptions all elements of $\underset{=}{A}$ and $\underset{=}{B}$, adding up to *N*^2^ + *M*·*N*, had to be determined. Typically, in GRNs there are far less connections than theoretically possible leading to a sparse matrix $\underset{=}{A}$. Regarding this property and avoiding problems occurring by the number of usually available measurement data (parameter identifiability, local or unique solutions, computational effort) the NetGenerator algorithm applies a heuristic approach as described in the next subsection.

### Heuristic multi-stimuli multi-experiment approach

The novel NetGenerator algorithm is a heuristic multi-stimuli and multi-experiment approach. The heuristic is based on the observation that in GRNs the number of connections is much lower than all possible connections. Further, since the applied stimulus is the cause of the observed dynamical changes, the network can be considered as a hierarchical structure originating from the input. The NetGenerator algorithm implements both observations by an iterative development of the state-space system (2) by including *coupled sub-models* for each time series based on a structure optimisation iteratively increasing the number of connections. Structural changes are taking place only if they result in a better adaptation of simulated to measured behaviour. The terms multi-stimuli and multi-experiment mean that the extended algorithm can handle more than one changed input and data of several experiments, respectively.

**Figure 2:**
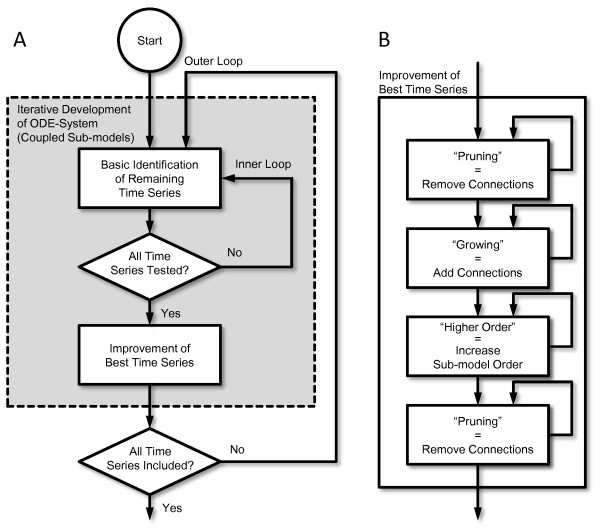
**NetGenerator work flow.** NetGenerator work flow displaying the main steps of the algorithm **(A)** and the improvement of best time series **(B)**. In the main work flow, the outer loop iterates over all state variables (sub-models), while in the inner loop the remaining time series are tested, i.e. a basic structure and parameters are identified. The best time series is improved further (“Growing”, “Higher Order”, “Pruning”) and included into the model as a state variable.

In Figure 2 (A) the main work flow of the algorithm is displayed. One outer loop, starting with empty $\underset{=}{A}$ and $\underset{=}{B}$, iterates over all sub-models (state variables) to which the measured time series should be linked. At the *i*th iteration step of the outer loop already *i* − 1 time series have been included in the model as sub-models. There are *N* − *i* + 1 remaining time series to be included. The *i*th state equation (sub-model) would be described by

(3) $${\dot{x}}_{i}=\underset{n\in N_{i}}{\sum }a_{i,n}x_{n}+\underset{m\in M_{i}}{\sum }b_{i,m}u_{m}$$

containing connections from state variables, *N*_*i*_ being the indices of state-state connections including the self-regulatory term *a*_*i*,*i*_*x*_*i*_, and connections from inputs with *M*_*i*_ being the indices of input-state connections for the considered state variable *x*_*i*_. That means that only the parameters of sub-models have to be identified.

Since the algorithm aims at a low number of parameters, i.e. small |*N*_*i*_| ≤ *N* and |*M*_*i*_| ≤ *M*, the inner loop starts with basic models for the remaining time series containing only self-regulation, one input term as well as connections from “fix” prior knowledge if available, see respective subsection. Those basic structures can be extended by further incoming connections (“growing”) from already included sub-models and further inputs. Every structural change requires a parameter identification of the active connections with respect to the considered time series, as will be explained later in the corresponding subsection. For every different set of parameters the resulting model needs to be simulated, that is the numerical solution of an initial value problem has to be found, as will be described later in another subsection.

The basic sub-model which reproduces one of the remaining time series best, is chosen for further improvement, for details see Figure 2 (B), and included into the model as a state variable. The most important structural improvements are 

•“Growing”: further connections added

•“Higher Order”: increase sub-model order

•“Pruning”: connections removed

In the improvement step “growing” is not restricted to connections from time series that are already included in the model. For describing the influence of time series that have not yet been included as sub-models, the corresponding measured and interpolated data are used as inputs. Those connections form global feedbacks in the final model.

The increase of the dynamical order within the description of a time series is realised by *r*−1* additional* equations or intermediate state variables leading to the following form:

(4) $$\begin{array}{cccc} \;{\dot{x}}_{i} & = & a_{i,i}x_{i}+\underset{n\in N_{i}\setminus \{i\}}{\sum }a_{i,n}x_{n}+\underset{m\in M_{i}\setminus \{i\}}{\sum }b_{i,m}u_{m}\\ \;{\dot{x}}_{i+1} & = & a_{i,i}x_{i+1}+x_{i}\\ & \vdots & & \\ {\dot{x}}_{i+r-1} & = & a_{i,i}x_{i+r-1}+x_{i+r-2} \end{array}$$

In this way the dynamics of a certain sub-model are described by an *r*th order integrator chain allowing for reproduction of processes with more complex time courses. It should be emphasised that by applying this approach the number of parameters is not increased but on the other hand the number of state variables becomes larger than the number of time series data. In that case only the state variable with the highest order in such a sub-model is to be compared to time series data. Still, for the sake of simplicity all following algorithmic procedures are described for first-order sub-models.

In terms of the iterative process of including sub-models the different elements of the *final* system matrix

(5) $$\underset{=}{A}=\left[\begin{array}{cccc} a_{1,1} & a_{1,2} & \cdots & a_{1,N_{s}}\\ a_{2,1} & a_{2,2} & & \\ \;\vdots & & \ddots & \\ a_{N_{s},1} & & & a_{N_{s},N_{s}} \end{array}\right]$$

describe forward, local feedback and global feedback connections. Elements below the main diagonal become forward connections, whereas the main diagonal elements $\frac{}{}a_{1,1},\ldots ,a_{N_{s},N_{s}}$ describe local feedbacks or self-regulations, while the elements above the main diagonal represent global feedbacks. From a biological point of view the local feedbacks describe different mechanisms including not only feedback regulation, but also the important process of mRNA-degradation.

All the previously described structural procedures and the corresponding parameter identification are controlled by *a-priori* defined settings and options of the algorithm. Some of them are balancing network complexity and error between measurement and simulation. For example, additional connections are rejected if they are not improving the objective function value to a significant extent while on the other hand connections are removed only if they are not worsening the result significantly. Further important options of the algorithm are explained in the respective subsection.

### Parameter identification

The parameter values of an active sub-model are identified by a non-linear optimisation, minimising the error between simulated and measured time series data of multiple experiments. The initial parameters required for this optimisation are obtained by a linear regression. For one specific first order state variable *x*_*i*_ equation (3) can be rewritten as

(6) $${\dot{x}}_{i}=\left[u_{1},\ldots ,u_{M_{i}},\;x_{1,}\ldots ,x_{N_{i}}\right]\cdot {\underset{-}{\theta }}_{i,\mathrm{init}}$$

with

(7) $${\underset{-}{\theta }}_{i,\mathrm{init}}={\left[b_{1},\ldots ,b_{M_{i}},\;a_{1},\ldots ,a_{N_{i}}\right]}^{T}$$

being the parameter vector of some of the elements of $\underset{=}{B}$ and $\underset{=}{A}$, respectively, as determined by structural optimisation using only a subset of inputs and state variables influencing the considered *i*th state variable. Satisfying the measured data in an optimal way the unknown parameters can be determined by the following equation of linear regression, see e.g. [13],

(8) $$\begin{array}{ccc} {\underset{-}{\theta }}_{i,\mathrm{init}} & = & {\left({\left[\begin{array}{cc} \underset{=}{U} & \underset{=}{X} \end{array}\right]}^{T}\;\underset{=}{W}\;\left[\begin{array}{c} \underset{=}{U}\;\underset{=}{X} \end{array},\;\right]\right)}^{-1}\\ & & \times \left({\left[\begin{array}{c} \underset{=}{U}\;\underset{=}{X} \end{array},\;\right]}^{T}\;\underset{=}{W}\;{\underset{-}{\dot{x}}}_{i,\mathrm{num}}\right) \end{array}$$

with the weight matrix $\underset{=}{W}$ and the state variable derivatives ${\underset{-}{\dot{x}}}_{i,\mathrm{num}}$. The latter are calculated by numeric differentiation of the respective time series (output) data. This means the vector ${\underset{-}{\dot{x}}}_{i,\mathrm{num}}$ is not the vector of state variables but the vector of time points of the considered time series derivatives. Its length (*T* = *K* + *K*_*interp*_) equals the sum of the number of measurement time points and interpolation time points, as outlined in the subsection on data pre-processing. The reason for the use of interpolated data is the avoidance of over-fitting. The different influence of measured and interpolated values is considered in the elements of the weight matrix $\underset{=}{W}$ possessing the dimensions *T* × *T*. Since the model must be valid for all *E* experiments, the respective input and time series data are *concatenated*, indeed resulting in $T=\sum T_{e}$, *e* = 1,…,*E*. This becomes possible because the regression approach implicitly assumes a “dynamic independence” of data points. The dimensions of the other variables are $\underset{=}{U}\;:\;T\times \left|M_{i}\right|$ and $\underset{=}{X}\;:\;T\times \left|N_{i}\right|$, with the number of rows of each matrix also equalling to the total number of time points. Both dimensions of $\underset{=}{U}$ reflect necessary algorithmic changes due to the consideration of multi-input multi-experiment data in this NetGenerator version, because |*M*_*i*_| > 1 represents multiple inputs while the concatenated data of length *T* considers multiple experiments. For the sake of completeness it should be mentioned that higher-order sub-models are initialised first by their first-order equation and then adapted such that total time constant and static gain remain the same.

The non-linear optimisation of the parameters for the *i*th sub-model, initialised by the solution of the linear regression (8), is based on the minimisation of the objective function (model error)

(9) $$J_{i,\mathrm{output}}=\overset{E}{\underset{e=1}{\sum }}\overset{T_{e,i}}{\underset{k=1}{\sum }}\left[w(t_{k})\cdot {\left(x_{e,i}(t_{k})-{\hat{x}}_{e,i}(t_{k},{\underset{-}{\theta }}_{i})\right)}^{2}\right]$$

describing the deviation between measured *x*_*e*,*i*_and simulated ${\hat{x}}_{e,i}$ time series at different time points *t*_*k*_ depending on the parameter vector ${\underset{-}{\theta }}_{i}$. The minimisation following (9) is an optimisation problem of the least squares type featuring a double sum of experiments *e* = 1,…,*E* and time points *k* = 1,…,*T*_*e*,*i*_. In contrast to the objective function applied in former NetGenerator versions, now *E* multiple experiments are considered. The simulated time series are compared to measured and also interpolated data weighed by different *w*(*t*_*k*_) avoiding over-fitting. A further weighing based on properties of the data, like for example the maximal range, is not necessary since the described pre-processing normalises and scales the data. For the optimisation problem, the new NetGenerator implementation applies the “L-BFGS-B” algorithm, [14], of the optim R-function, which has the ability to solve bounded non-linear optimisation problems.

### Consideration of prior knowledge

For improving the results, prior knowledge about the network connections can be integrated into the network inference. This version of NetGenerator provides two modes for integration of prior knowledge about connections of stimuli on time series as well as between the time series: (i) “fix” and (ii) “flexible”. For both modes the knowledge can be provided in form of connection matrices ${\underset{=}{A}}_{\text{fix—flexible}}$ and ${\underset{=}{B}}_{\text{fix—flexible}}$ resembling the system matrix and input matrix, respectively, as well as additional matrices containing reliability scores of the connections. The connection matrices can contain single-valued information about connection (1), no connection (0), activation (10) and inhibition (−10). Fix integration represents rigid model requirements that cannot be ignored by the heuristic. Therefore fix connections are always included in the model structure.

Flexible integration allows the inference heuristic to ignore prior knowledge when the model fit is substantially worsened. This is represented by an additional term in the objective function (model error) now resulting in

(10) $$J_{i}=J_{i,\mathrm{output}}+\lambda \left[\underset{j\in N_{i}}{\sum }s_{i,j}^{A}d_{i,j}^{A}+\underset{k\in M_{i}}{\sum }s_{i,k}^{B}d_{i,k}^{B}\right].$$

The term *J*_*i*,*output*_ corresponds to the previously in (9) defined evaluation of output deviation, while *λ* weighs the overall consideration of prior knowledge, *s* represent the score values of the respective prior knowledge and *d* describe the distances between the prior knowledge and the modelled structure (incoming connections) evaluated by comparison of signs. That means the resulting elements of $\underset{=}{A}$ and $\underset{=}{B}$ are converted into the described notation of 0, 1, 10, and −10, thus permitting a comparison with elements of flexible prior knowledge connection matrices. Here we consider two types of prior knowledge *origin*: (i) gene interactions automatically extracted from published literature and (ii) predicted transcription factor binding sites (TFBS) in the proximal promoter region of target genes.

For the extraction from published literature the software Pathway Studio V9 provides a gene relation database termed ResNet Mammalian, which has been compiled by automatic extraction of interactions from PubMed, as evaluated by [15]. As shown in the latter publication, gene relations derived from Pathway Studio V9 can be considered of high quality, since in general scientific literature is a reliable resource and the false positive rate is reported to be about 10 *%*.

Further, the tool matrix-scan from the RSAT toolbox determines putative TFBS in the promoter regions of target genes, which might be involved in transcriptional regulation [16]. This approach requires known sequence motifs of the investigated transcription factors as well as promoter sequences. Sequence motifs are stored in form of position weight matrices (PWM), which describe relative nucleotide frequencies for each motif position, as can be obtained from the Transfac database (Version 2010) [17]. Gene promoter sequences are available from Ensembl using biomaRt, [18].

Additional prior knowledge about the regulatory potential of the individual genes can be obtained by examining the known molecular functions. For example, the interaction between genes coding for non-regulatory proteins, such as structural proteins, and target genes can be assigned “no connection”.

### Simulation and graphical output

For every comparison of measurement and simulation as well as the generation of results the model equations (2) must be integrated. This corresponds to an initial value problem that is solved numerically. Since the recent implementation of the NetGenerator algorithm is in R, repeated operations of certain types take a long time. Therefore, the model itself is implemented in C, created iteratively and simulated applying the implicit method “impAdams” of the R-package deSolve, [19]. The necessary initial conditions ${\underset{-}{x}}_{0}=\underset{-}{x}(t_{0})$ are either measurement data or extrapolated measurement data typically at *t*_0_ = 0 of the respective time scale.

The final result of the NetGenerator algorithm is a parametrised model of the considered GRN. Moreover, the new implementation of the algorithm contains important graphical output facilities which have been extended to meet the needs of displaying multi-input multi-experiment data as well as different results concerning prior knowledge. First, there is a graphical comparison of measurements and simulations, showing the single measured data points and the corresponding simulated trajectory. This can be done either by comparison of each component (gene) over all experiments or by displaying the data for each experiment independently. Second the resulting network structure can be displayed as a directed graph applying the language DOT and the software collection Graphviz, [20]. Nodes denote the biochemical components, e.g. genes, and edges display connections of either activation or inhibition. In case of applying prior knowledge (see respective subsection), a comparison between the inferred network and this knowledge is displayed with a colour code. Black edges denote inferred connections without prior knowledge, green edges present an agreement, red edges could either have a wrong sign (e.g. activation instead of inhibition) or be connections that do not comply with prior knowledge, while grey dashed edges stand for prior knowledge not reproduced in the inferred network.

### Further settings and updated methods

The NetGenerator algorithm itself can be controlled by parameters (settings) and also contains further methods that will be summarised in the following. An important setting is the “allowedError” that controls the structure optimisation. If the objective function value of a certain sub-model structure is worse than this value the model structure must be extended as described. Therefore smaller values of “allowedError” are indirectly leading to more complex structures. Further important settings are the maximal number of connections and sub-model order.

Additional updated or new methods, not described extensively here, include non-linear modelling and knowledge-based methods. The optional non-linear modelling approach contains an additional sigmoid transformation of the linear combination described in this publication. This transformation has its biological background in the saturating behaviour of gene expression. The additional non-linear parameters of each sub-model are determined by the described non-linear parameter identification, too. Amongst further knowledge-based methods, the most important presents the possibility of retrieving network information from databases and combining this information with the inferred model in a directed graph. In that way, the biological interpretation can be extended by introducing unmeasured components into the network structure.

### Availability

The algorithm has been implemented as a package in the programming language / statistical computing environment R, [9]. It is available in form of a testing bundle containing both the algorithm and the examples at http://www.biocontrol-jena.com/NetGenerator/NetGeneratorBundle.zip

## Results

### Example networks

We applied the NetGenerator algorithm, which has been described extensively in the Methods section, to 3 benchmark examples and 1 real-world example to examine the performance of our approach. At first, we consider the three benchmark systems, their corresponding artificial data and inferred networks in order to test the reliability and performance of our algorithm. Particularly, we investigated whether network inference from multiple data sets, originating from different stimulation experiments, is beneficial. Finally, we applied NetGenerator to microarray time series data gained from human mesenchymal stem cells. We focussed on the modelling of gene regulation that occurs during *in vitro* stimulation of chondrogenic differentiation of these cells, with emphasis on the different effects triggered by multiple stimuli in the inferred model.

### Benchmark examples

We constructed three fully parametrised benchmark systems based on linear time-invariant descriptions, i.e. they are composed of differential equations representing the time series of genes and two external stimuli (*u*_1_ and *u*_2_). The systems are characterised by a different degree of cross-talk between the components with respect to the external stimuli, that is “full cross-talk” (FCT): all components are influenced by all stimuli, “limited or low cross-talk” (LCT): some of the components are influenced by more than one stimulus, and “no cross-talk” (NCT): the stimuli influence distinct components resulting in separate networks. They also differ in the number of genes (FCT: 5, LCT: 4, NCT: 7) and the parameters. The artificial data were generated exhibiting characteristics of real microarray time series data, i.e. low number of time points (six), exponentially increasing time intervals, and additional normally distributed noise $N(0,\;0.05^{2})$. In summary, this procedure led to sample data sets containing matrices with number of rows equalling number of genes and six columns (time points).

*Evaluation measures.* The network inference of benchmark systems can be evaluated by determining the final objective function value (model error) *J* according to equation (10), the computation time *t*_*C*_, and statistical measures that quantify the performance of the network inference by comparing the known structure with the inferred structure. The indicated computation times resulted from running the examples on a x86-PC with a 2*.*33 *GHz*CPU. The measures comprise sensitivity (SE), specificity (SP), precision (PR) and *F*-measure (FM). The definitions of the measures take into account the correctly integrated edges (true positives, TP), the falsely integrated edges (false positives, FP), the truly missing edges (true negatives, TN) and true edges that are not contained in the model result (FN). False positives (FP) were further grouped into *FP*_*s*_, connections integrated with wrong sign and *FP*_*n*_, modelled interactions which are not present in the real network. This leads to the following definitions:

$$\begin{array}{ccc} \mathrm{SE} & = & \mathrm{TP}/(\mathrm{TP}+\mathrm{FN}+FP_{s})\\ \mathrm{SP} & = & \mathrm{TN}/(\mathrm{TN}+FP_{n})\\ \mathrm{PR} & = & \mathrm{TP}/(\mathrm{TP}+FP_{n}+FP_{s})\\ \mathrm{FM} & = & 2\cdot \mathrm{PR}\cdot \mathrm{SE}/(\mathrm{PR}+\mathrm{SE}) \end{array}$$

For all three benchmark examples, we evaluated the inference by those statistical measures showing the reproduction of the system structure and time series by the model.

*FCT scenarios and network inference evaluation*. For FCT, artificial data generation and subsequent network inference was performed within three scenarios: (i) “*S*_1_”: single experiment applying only *u*_1_, (ii) “*S*_2_”: single experiment applying only *u*_2_ and (iii) “M”: multiple experiment integrating experiments “*S*_1_” and “*S*_2_”. For the special case of FCT, the scenarios allowed us to directly compare the inference of multiple stimuli data sets with the inferences of single stimulus data sets.

**Figure 3:**
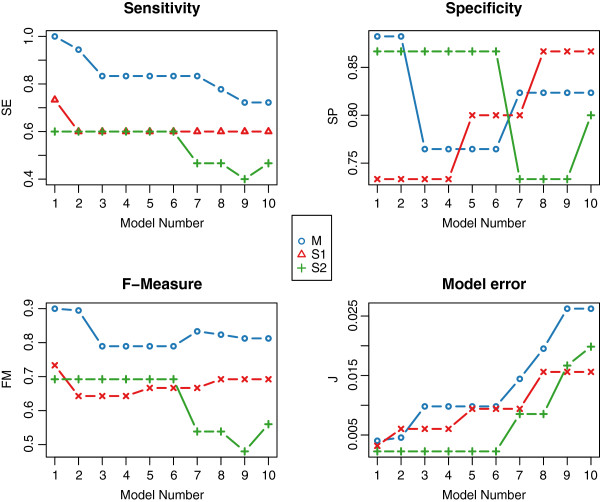
**“Full cross-talk” example: inference statistics.** Comparison of different NetGenerator inference results (varying the “allowedError” parameter) of three “full cross-talk” (FCT) scenarios (“M”, “S_1_”, “S_2_”) using three statistical evaluation measures (sensitivity, specificity, and F-measure) and the model error.

We applied the network inference to each of the three scenarios (“M”, “*S*_1_”, “*S*_2_”) for a series of 10 different settings varying the previously described “allowedError” = 0*.*001, 0*.*002, …, 0*.*01 resulting in 10 models, see Figure 3. Results for all statistical measures are depicted as connected points in individual boxes. The three scenarios are plotted in distinct colours (“M”: blue, “*S*_1_”: red, “*S*_2_”: green) in each box. With regard to sensitivity, M models performs best, showing gradually decreasing values. Specificity obtains highest values for the first and second model. *F*-measure results, which benefit from high sensitivity values, display good performance for all M models. The resulting model error increases gradually as expected, due to the increased “allowedError”, which is defined per time series. Analysing these results, we found “M 1” (*TP* = 18, *TN* = 15, *FP*_*n*_ = 2, *FP*_*s*_ = 0, *FN* = 0) to be optimal with respect to the evaluation measures (*SE* = 1, *SP* = 0*.*88, *FM* = 0*.*94, *J* = 0*.*004). For this model, the computation time was *t*_*C*_ = 92 *s*.

**Figure 4:**
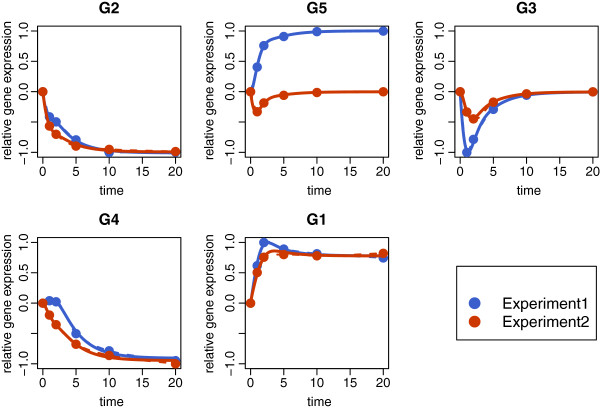
**“Full cross-talk” example: time courses.** Comparison of the “full cross-talk” (FCT) time courses. Each panel displays the results of one gene: the simulated time course (solid line), interpolated measurements (dashed line) and the measured time series (dots) for both data sets (Experiment1 and Experiment2).

**Figure 5:**
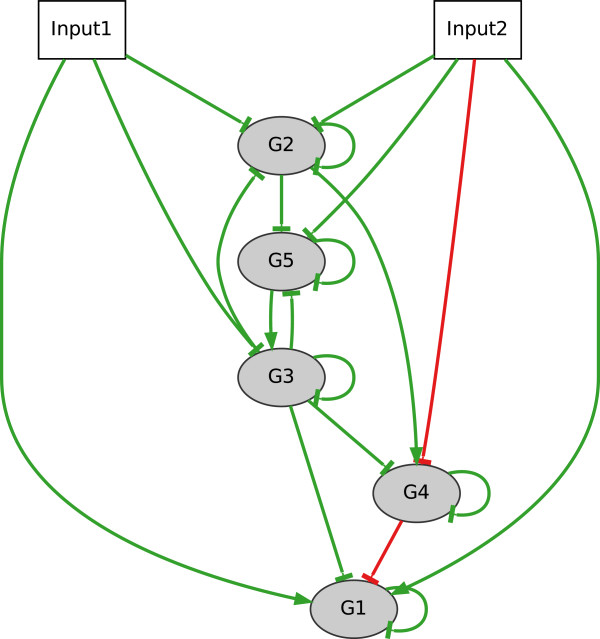
**“Full cross-talk” example: inferred network.** Network structure of the “full cross-talk” (FCT) model containing two simulated inputs (Input1, Input2) and five gene nodes. Inferred connections are highlighted in green (TP = 18), red (FP_*n*_=2) and dashed grey (FN = 0).

Dynamics of this model are displayed in Figure 4 showing a good reproduction of all time series for each of the two experiments. In Figure 5, the corresponding regulatory network is presented in form of a directed graph. Here, the colour code is not denoting a reproduction of prior knowledge but a graphical means displaying TP (green), FP (red) and FN (grey/dashed) connections.

**Figure 6:**
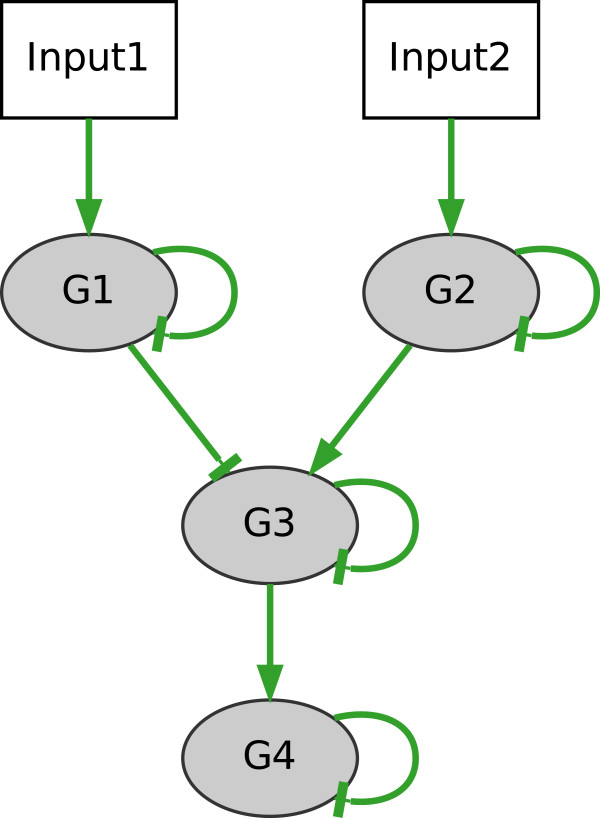
**“Limited cross-talk” example: inferred network.** Network structure of the “limited cross-talk” (LCT) model containing two simulated inputs (Input1, Input2) and four gene nodes. Inferred connections are highlighted in green (TP = 9), red (FP_n_ = 0) and dashed grey (FN = 0).

**Figure 7:**
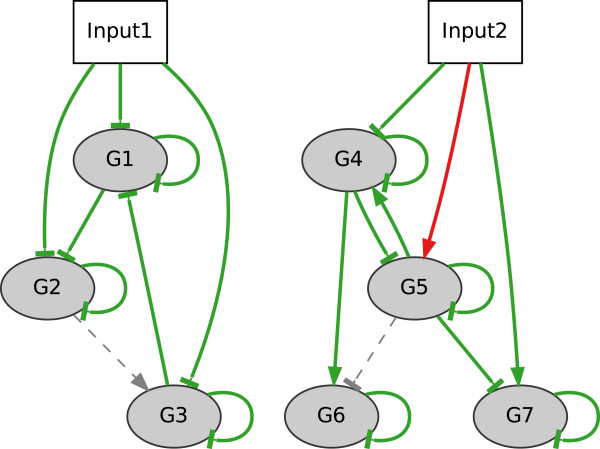
**“No cross-talk” example: inferred network.** Network structure of the “no cross-talk” (NCT) model containing two simulated inputs (Input1, Input2) and seven gene nodes. Inferred connections are highlighted in green (TP = 18), red (FP_n_ = 1) and dashed grey (FN = 2).

*LCT and NCT network inference evaluation*. In order to test whether NetGenerator is capable of inferring different cross-talk structures, we generated benchmark systems LCT and NCT. Both contain biologically motivated types of cross-talk, such as cross-talk of downstream components or separate sub-networks (no cross-talk). Inference of both networks was successful, shown by high statistical measures (*SE*_*LCT*_ = 1, *SP*_*LCT*_ = 1, *FM*_*LCT*_ = 1, *J*_*LCT*_ = 0*.*0007, *SE*_*NCT*_ = 0*.*9, *SP*_*NCT*_ = 0*.*98, *FM*_*NCT*_ = 0*.*92, *J*_*NCT*_ = 0*.*003), the inferred network structures in Figure 6 and Figure 7, and the good reproduction of the time courses (Additional file 1 and Additional file 2). The computation time for inference of LCT and NCT was *t*_*C*_ = 28 *s* and *t*_*C*_ = 33 *s*, respectively.

### Chondrogenesis model

*Background of chondrogenic data*. Human mesenchymal stem cells (hMSC) are multi-potent adult stem cells that have the capacity to differentiate into a variety of cell types depending on the external stimulus, [2]. Regulation of lineage-specific genes is crucial in this temporal process, [21]. Transforming growth factor (TGF)-beta1 is essential for induction of chondrocyte differentiation of hMSC, a process which is strongly enhanced by the additional presence of bone morphogenetic protein (BMP)2, [22] and [23]. In this section, we describe the complementary effects of TGF-beta1 and BMP2 by multi-stimuli multi-experiment inference applying the NetGenerator algorithm.

*Microarray time series data*. hMSC from bone marrow were commercially obtained (Lonza) and cultured as described in [2]. To induce chondrogenic differentiation trypsinised hMSC were pelleted and subsequently incubated in culture medium supplemented with 100 *nM* dexamethasone, 10 *ng*/*mL* TGF-beta1 and, if applicable, 50 *ng*/*mL*BMP2. Time-dependent gene expression was studied under three experimental conditions: (i) following treatment with TGF-beta1 (“T”), (ii) following treatment with TGF-beta1 + BMP2 (“TB”) and (iii) untreated hMSC as a control. At 10 different time points (0, 3, 6, 12, 24, 48, 72, 128, 256, 384) *h*after addition of the stimuli, RNA was isolated from three technical replicates per time point and measured on Affymetrix HG-U133a microarrays.

*Pre-processing and filtering*. Raw microarray data was pre-processed as described in the corresponding sub-section. This included the use of custom chip definition files provided by [10] and application of the RMA method [11]. This procedure resulted in logarithmised gene expression estimates for 12 095genes.

Modelling a small-scale GRN using microarray data requires adequate filtering of genes. We tested all genes for differential expression, used functional annotation and expert knowledge. Differentially expressed genes were identified for both experiments (“T”, “TB”) by computing adjusted *p*-values using LIMMA. All genes with an adjusted *p*-value less than 10^−10^and an absolute fold-change greater than 2 for any time point were considered significant. Using those criteria, 192 genes were found to be differentially expressed compared to control as well as between “T” and “TB”. Subsequently, we selected from this group 10 annotated transcription factors (GO:0003700, sequence-specific DNA binding transcription factor activity) and associated 5 of them (SOX9, MEF2C, MSX1, TRPS1, SATB2) with our investigated process (GO:0051216, cartilage development). Those genes may be involved in promoter-dependent regulation, which is important for binding site predictions. Furthermore, we added COL2A1, ACAN, COL10A1, all three essential marker genes of chondrocyte differentiation, which encode essential structural proteins of the extracellular matrix.

*Prior knowledge*. Prior knowledge was taken into account as described in the corresponding sub-section. Gene interactions were retrieved from the Pathway Studio ResNet Mammalian database. We obtained 6 gene-gene and 5 input-gene regulatory interactions. Gene-gene interactions were passed as flexible prior knowledge to NetGenerator. Input-gene interactions were not integrated. Additionally, potential gene interactions were determined by binding site predictions. For this purpose, we obtained PWMs for SOX9, MEF2C and MSX1 from the Transfac database and promoter sequences 1000 *bp* upstream from the transcription start site. Both PWMs and sequences were loaded into matrix-scan from RSAT, which is performed with default options (weight-score >1, *p*-value <10^−4^) and organism-specific estimation of background nucleotide frequencies. The resulting significant binding sites have been listed in the table of Additional file 3. The observed high significance of all matches minimises the risk obtaining similar results from random sequences.

*Network inference of multi-stimuli (TGF-beta1 and BMP2) multi-experiment data*. After pre-processing, the input and time series data of the microarray experiments were passed to NetGenerator for automatic network inference. According to the experimental set-up, the available data sets describe two experiments: only TGF-beta1 stimulation (“T”) and TGF-beta1 + BMP2 stimulation (“TB”). This is mirrored by the two distinct input data matrices both describing the respective stimuli by step functions

$${\underset{=}{U}}_{T}=\left[\begin{array}{cc} 1 & 0\\ 1 & 0\\ \vdots & \vdots \\ 1 & 0 \end{array}\right],\;\;\;\;{\underset{=}{U}}_{\mathrm{TB}}=\left[\begin{array}{cc} 1 & 1\\ 1 & 1\\ \vdots & \vdots \\ 1 & 1 \end{array}\right]$$

**Figure 8:**
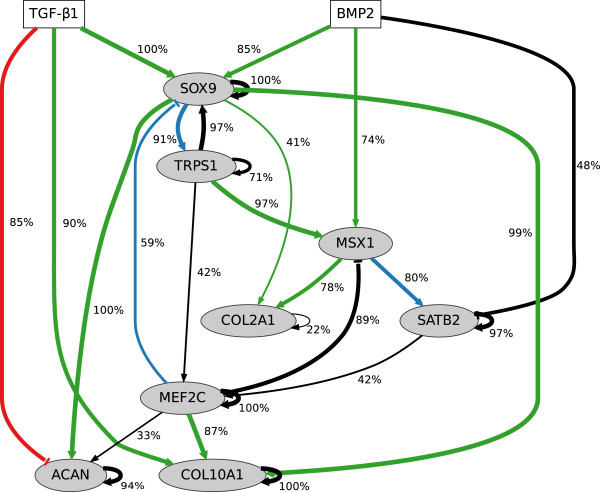
**Chondrogenesis system: inferred network.** Network of the chondrogenesis system, which contains two inputs (TGF-beta1 and BMP2). Nodes represent either transcription factor genes (SOX9, MEF2C, MSX1, TRPS1, SATB2) or genes coding for structural proteins (COL2A1, COL10A1, ACAN). Connections are coloured in green (consistent with prior knowledge), red (contrary to prior knowledge), blue (predicted connection with existing binding site) and black (predicted interaction). Connection widths and percentage labels illustrate the frequency of occurrence in the validation procedure.

*Model evaluation and validation*. The inference results of the chondrogenic system, the GRN and the graphical comparison of time series, are displayed in Figure 8 and Additional file 4, respectively. The resulting network contains 20 connections: 14 gene-gene connections and 6 input-gene connections. Compared to the prior knowledge, there are 10 green connections (consistent), 1 red connection (wrong sign) and 3 blue connections (additional colour code for predicted binding site).

For validation, we performed resampling which is based on random perturbation of time series data. A Gaussian noise component $N(0,\;0.05^{2})$ was added to the time series data which is used for subsequent model inference. Repeated performance (100×) led to a series of inference results as well as relative frequencies for each of the connections of the nominal model, i.e. the proportion of models containing that specific connection. Those frequencies imply a reliability ranking of all nominal connections. Most of the connections were inferred with high frequency, (76±24) *%*, see Figure 8. Particularly, this applies to connections which reflect prior knowledge. Also, inferred connections which are associated with a predicted binding site (blue colour) were present in more than 50 *%* of the models.

*Network interpretation*. SOX9 exhibits a central role in this chondrogenic network and is activated by both TGF-beta1 and BMP2. This indicates a complementary effect of both stimuli on the expression of SOX9. Activated SOX9 drives the expression of its target genes COL2A1, ACAN and COL10A1 [24-26]. This regulation marks the essential formation of cartilage-specific structural components of the extra-cellular matrix and the differentiation of hMSC towards chondrocytes. Beside this process, SOX9 also activates the repressor gene TRPS1 and vice versa. Regulatory interactions between both factors has not yet been addressed in the literature. Additionally, the SOX9 binding motif is present in the proximal promoter of the TRPS1 gene according to the prior knowledge. There is also a modelled effect from TRPS1 on MEF2C, which in turn activates COL10A1 and ACAN, but represses SOX9. This represents a negative global feedback from MEF2C on SOX9 in our model. MEF2C also represses the expression of MSX1, which is solely activated by BMP2 stimulus and activates COL2A1 according to the prior knowledge [27]. MSX1 also activates the SATB2 gene, which in turn activates MEF2C expression. Negative regulation of ACAN by TGF-beta1 is contrary to prior knowledge, as indicated by the red connection of the network graph. However, TGF-beta1 can also activate ACAN indirectly through SOX9, [24]. In summary, the central player SOX9 is influenced by both TGF-beta1 and BMP2. Essential structural proteins are not solely regulated by SOX9, but also by other transcription factors (MEFC, MSX1). Moreover, SOX9 and MSX1 are repressed by MEF2C through negative feedback that involves TRPS1 and SATB2.

## Discussion

The NetGenerator algorithm for automatic network inference from multi-input multi-experiment time series data and prior knowledge, described in the methods section, will be classified and distinguished from other methods in the next sub-section. Therefore, its properties will be reviewed and justified showing advantages and disadvantages to other approaches. Our discussion contains a wide spectrum of other methods, but will only go into detail for the ones closely related to NetGenerator. Also, further specifications of NetGenerator will be summarised without a detailed comparison to other methods.

### Classification of the algorithm

Good review articles on methods for automatic inference of GRNs can be found in [1,3,4]. The different methods can be classified by the data type (static or dynamic), the mathematical approach (e.g. probabilistic vs. deterministic) and the result (e.g. undirected vs. directed graphs, algebraic correlation vs. dynamic models) whereby various combinations are possible. Mutual information methods (for a review of ARACNE, CLR and MRNET see [28]) are based on evaluating the statistical dependencies of large data sets resulting in undirected graphs. In comparison to NetGenerator they possess far different preconditions and purposes, for example they do not consider a concerted influence of the variables or the dynamics of the state-space concept, and therefore a more detailed comparison is set aside. Even though dynamical Boolean networks for gene-regulation, first proposed by [29], possess some similarities to discrete-valued state-space models, their rule-based approaches typically lead to rather qualitative results (for an overview of recent methods see the aforementioned review articles).

Very often, like in case of the core elements of NetGenerator, GRNs are based on linear modelling, i.e. the behaviour of one variable depends on a linear combination of other variables. Still the method can be a combination of either probabilistic or deterministic approach as well as algebraic correlation modelling (equations system) or dynamic modelling (differential equations system). In the case of the probabilistic modelling which is especially covered by static and dynamic Bayesian networks (see aforementioned review articles) the inference is based on the application of probability distributions to describe the uncertainties or noise inherent in GRNs. Beside the differences in the mathematical approach, probabilistic modelling includes the determination of statistical parameters and therefore generally more data replicates are required in comparison to deterministic modelling approaches such as NetGenerator.

Deterministic linear modelling applied to automatic network inference, [30], can be distinguished into at least two types depending on the results: (i) algebraic equations systems, e.g. [31], and (ii) differential (difference) equations systems, e.g. [32]. Although they have different prepositions on the dynamics of time series data, both types can be solved by linear regression. Still, there is a disproportion between the number of free parameters and available measurement data on the one hand and the property of sparsity of GRNs on the other hand. For the former interpolated data points can be applied under the assumption that the influence of the chosen interpolation on the results can be neglected. For the reproduction of sparse networks the regression can be combined with model reduction, for example using the large group of LASSO-based algorithms, see e.g. [33-36], on the basis of PCA (SVD), [37], or a combination of both, [38]. For further approaches, see the aforementioned review articles.

In contrast to all these methods, we propose the NetGenerator algorithm dealing with the problem of data number and sparsity in a different way. The algorithm is not inferring the network structure and parameters in one go. Instead we applied an heuristic approach of explicit structure optimisation, which iteratively generates a system of sparsely coupled sub-models. In that way, the GRN property of possessing more or less hierarchical input to output structures is reproduced. Thus, only the parameters of sub-models describing one time series have to be determined. A major drawback of regression-based solutions of linear differential (difference) equations systems is the necessity of applying numerical derivatives of small sample size and noisy data, which have a strong influence on the resulting network and modelled dynamics. NetGenerator uses a different solution, whereby the regression just provides initial parameters for a non-linear optimisation of an objective function of the least squares type. Overall, the final dynamic network can be obtained by a lower computational effort, because in comparison to the total number of parameters (*N*^2^ + *M*·*N*) in the model description (2) only a small number of parameters has to be determined.

### Inference from multi-stimuli multi-experiment time series data

The concept of inferring from multiple data sets is also applied by [38], however on the basis of principal components of those data sets. The work of [39] provides a multiple methods framework to integrate distinct types of data like steady-state and time series data, focussing mainly on the combination of knock-out and stimulation data.

The proposed NetGenerator V2.0 algorithm allows for integrating data sets of multiple experiments with multiple stimuli. In the inferred models, weighed input terms represent external stimuli and resulting GRNs represent the merged effects of the diverse experiments. Therefore, from a biological point of view, the algorithm is able to handle experiments which investigate the degree of cross-talk.

We applied and tested this feature for 3 benchmark examples and 1 real-world example, the gene regulation during chondrogenic differentiation. The evaluation of the benchmark examples’ results showed the power of the algorithm to infer the network structure and to reproduce the time series. Further, for a special system of “full cross-talk”, i.e. all components are influenced by all stimuli, we could show that the simultaneous utilisation of different data sets leads to higher model quality compared to modelling data sets individually. The reason for this effect is due to the different stimulation by another external input which alters the time series data qualitatively and quantitatively, something that could not be achieved by biological replicates of a single input experiment. This underlines the benefit of using our integrated approach. Further, the presented examples LCT and NCT are possible outcomes of GRN investigations. In the first case, there are two different types of genes: some are induced by one stimulus only and some are induced by multiple stimuli. The model inferred by NetGenerator contains both the separate and common structural elements. The special case of NCT occurs, if network parts are stimulated that are not connected at all. In summary, the extended NetGenerator takes advantage of multi-stimuli multi-experiment data by network refinement and extension.

We further inferred a two-stimulus network for hMSC differentiating towards chondrocytes. This network model contains gene regulatory events following the stimulation with two distinct chondrogenic factors, therefore providing a view on how genes involved in differentiation might be controlled by external molecules. Applying a subsequent resampling gives further information about the connections of this inferred GRN: (i) the majority of connections, especially the ones of prior knowledge and predicted binding sites, occur with a high frequency which can be considered a measure of reliability and (ii) the ranking of the frequencies can be used in interpreting the results with regard to biological hypotheses. Overall, this shows the importance for an extension of NetGenerator to deal with multiple data sets.

### Consideration of prior knowledge

The means to integrate prior knowledge (fix and flexible) into the network inference is a distinctive feature of the extended NetGenerator algorithm achieved by modifying the objective function. This feature can reduce the complexity of the structure optimization, although it strongly depends on the origin and quality of the given knowledge, see e.g. [7]. Using prior knowledge for network inference can also be found in several other algorithms, see [1,3,4].

For our example of chondrogenic differentiation, we exemplarily showed network inference using flexible prior knowledge about regulatory interactions extracted from a database (Pathway Studio). The graphical evaluation of the inferred network showed very good reproduction of the proposed prior knowledge. Further predicted connections could be associated with potential regulatory binding sites generated from sequence data (Transfac, Ensembl).

### Further aspects

Apart from the linear modelling presented in detail, the ability of NetGenerator to infer a non-linear model has been mentioned as a further option. The additional sigmoid function describing saturation in gene-expression has been proven successful before, e.g. [40-42]. Since the sigmoid transformation has also been used for neural network models, those inference methods are sometimes classified as such.

Besides the many advantages and possible application areas, there are minor restrictions of NetGenerator: it should be applied to pre-processed data without high correlations, it infers networks from measured time series data and due to the heuristic approach it cannot be proven that the global solution was found. The latter can be improved by decreasing the influence of noisy data using a bootstrap (resampling) approach, see chondrogenesis example and [1]. One feature which might be introduced in subsequent versions is the application of “interventional” multi-experiment data, i.e. data originating from perturbations within the system. This can be dealt with by applying either experiment-wise prior knowledge or an additional module in the structure optimisation explicitly dealing with that kind of data.

## Conclusions

We presented the novel NetGenerator algorithm for automatic inference of GRNs, which applies multi-stimuli multi-experiment time series data and biological prior knowledge resulting in dynamical models of differential equations systems. This heuristic approach combines network structure and parameter optimisation of coupled sub-models and takes into account the biological properties of those networks: indirect transcriptional events for information propagation, limited number of connections and mostly hierarchical structures. The analysis of benchmark examples showed a good reproduction of the networks and emphasised the biological relevance of inferred networks with a different degree of cross-talk. The ability to infer a real-world example based on multi-stimuli multi-experiment data was shown by application of NetGenerator to a system of growth factor-induced chondrogenesis.

## Competing interests

The authors declare that they have no competing interests.

## Authors’ contributions

MW and SGH drafted the manuscript. SGH and DD contributed to the development and programming of the NetGenerator algorithm and software as well as to the mathematical and modelling background. MW and RG contributed to data processing, application of NetGenerator to examples, statistical evaluation and the biological interpretation. SV contributed to the generation of the benchmark systems and their artificial data. EJvZ contributed to experimental set-ups, measurements and biological interpretation of the chondrogenic investigation. All authors read and approved the final manuscript.

## Supplementary Material

Additional file 1 **Figure: “Limited cross-talk” example, time courses.** Comparison of the “limited cross-talk” (LCT) network time courses. Each panel displays the results of one gene: the simulated time course (solid line), interpolated measurements (dashed line) and the measured time series (dots) for both data sets (Experiment1 and Experiment2). Click here for file

Additional file 2 **Figure: “No cross-talk” example, time courses.** Comparison of the “no cross-talk” (NCT) network time courses. Each panel displays the results of one gene: the simulated time course (solid line), interpolated measurements (dashed line) and the measured time series (dots) for both data sets (Experiment1 and Experiment2). Click here for file

Additional file 3 **Table: Results of RSAT.** Results of RSAT matrix-scan tool using Transfac PWMs and genomic DNA sequences from Ensembl. Each row represents a predicted binding site with Transfac motif (“PWM”), target gene, start and end coordinates, the matched sequence, match score (“Weight”) and associated *p*-value. Click here for file

Additional file 4 **Figure: Chondrogenesis system, time courses.** Comparison of the chondrogenesis system time courses. Each panel displays the results of one gene: the simulated time course (solid line), interpolated measurements (dashed line) and the measured time series (dots) for both data sets (“T” and “TB”). Click here for file
