# Supplementary figures and images for: Inference of dynamical gene-regulatory networks based on time-resolved multi-stimuli multi-experiment data applying NetGenerator V2.0
Source: BMC Syst Biol. 2013 Jan 2;7:1. doi: 10.1186/1752-0509-7-1 (PMC3605253; doi:10.1186/1752-0509-7-1)

**G2**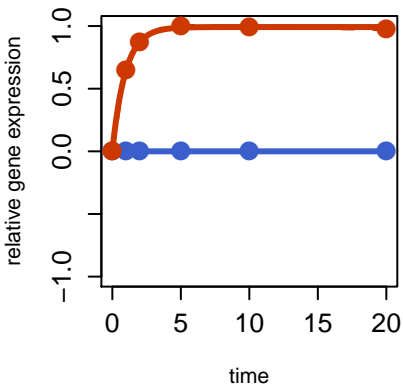**G1**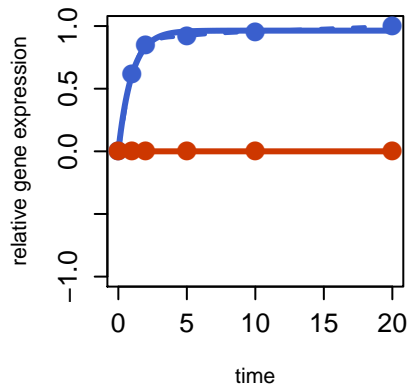**G3**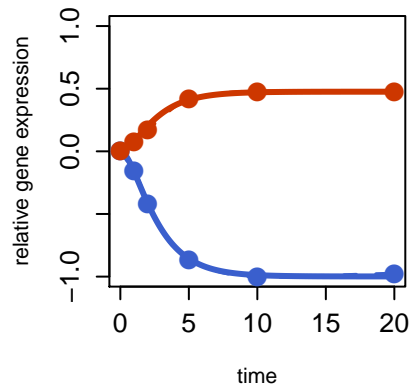**G4**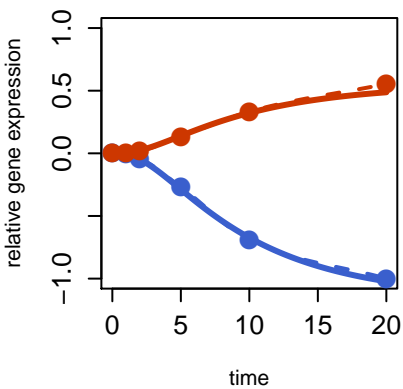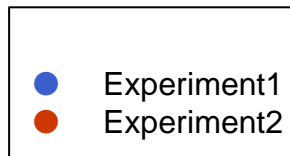

Supplement: Additional file 1 — Figure: “Limited cross-talk” example, time courses. Comparison of the “limited cross-talk” (LCT) network time courses. Each panel displays the results of one gene: the simulated time course (solid line), interpolated measurements (dashed line) and the measured time series (dots) for both data sets (Experiment1 and Experiment2). [file 1752-0509-7-1-S1.pdf]

**G3**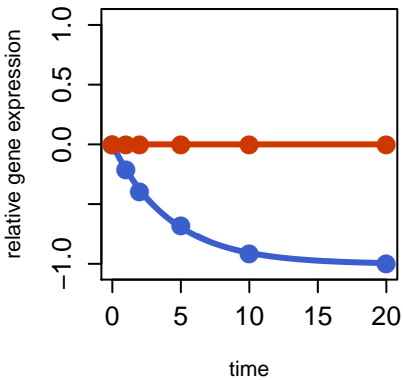**G1**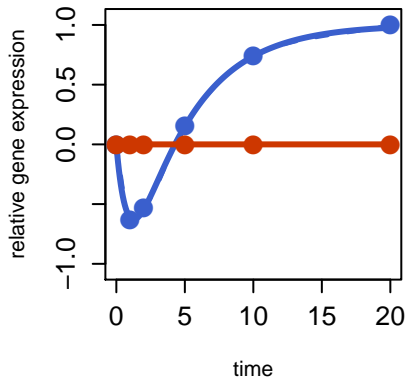**G2**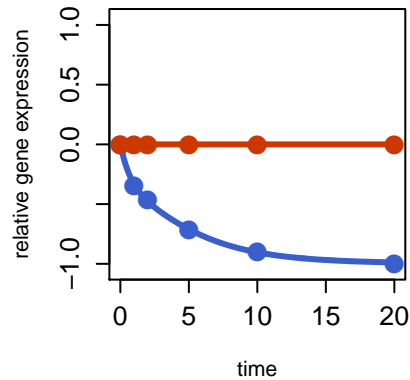**G6**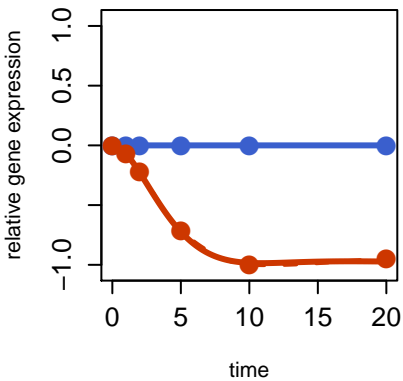**G4**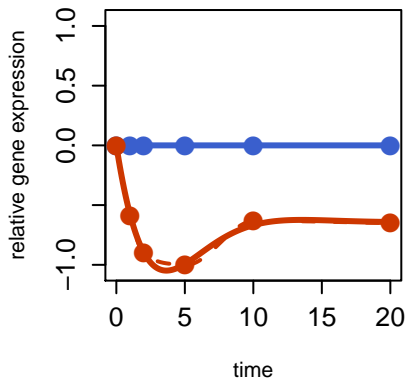**G5**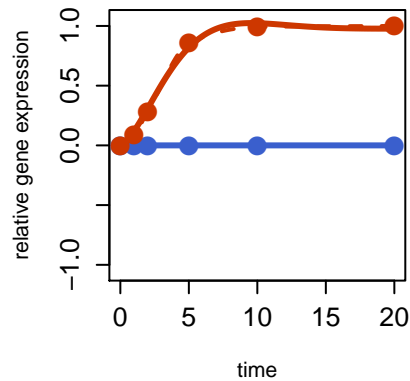**G7**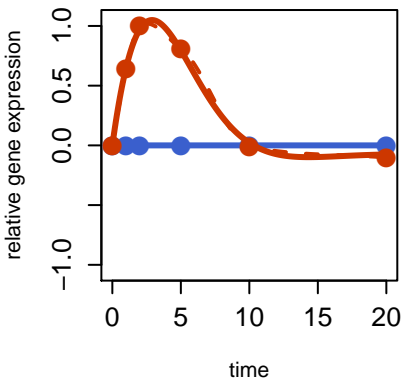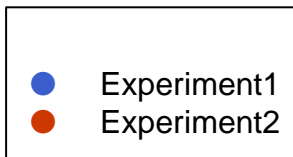

Supplement: Additional file 2 — Figure: “No cross-talk” example, time courses. Comparison of the “no cross-talk” (NCT) network time courses. Each panel displays the results of one gene: the simulated time course (solid line), interpolated measurements (dashed line) and the measured time series (dots) for both data sets (Experiment1 and Experiment2). [file 1752-0509-7-1-S2.pdf]

**TRPS1**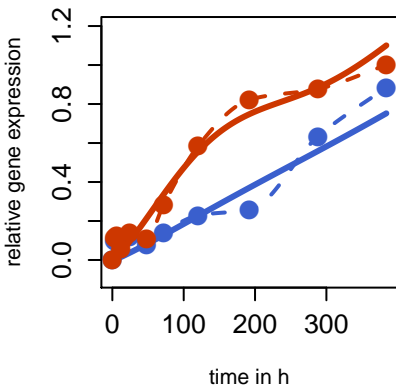**MEF2C**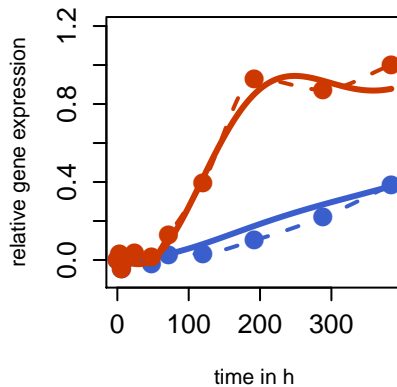**SOX9**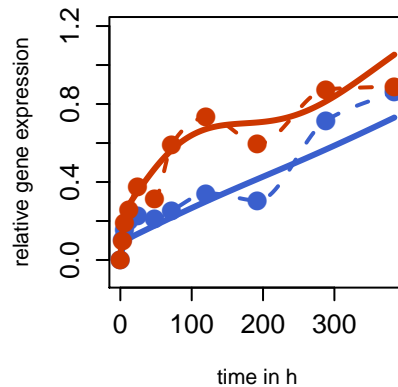**ACAN**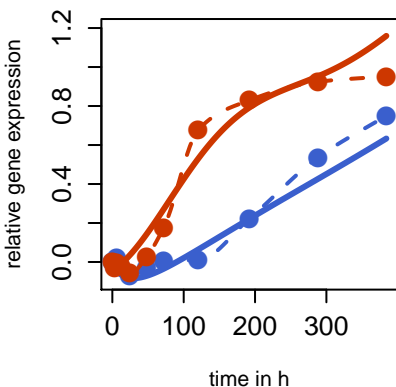**COL10A1**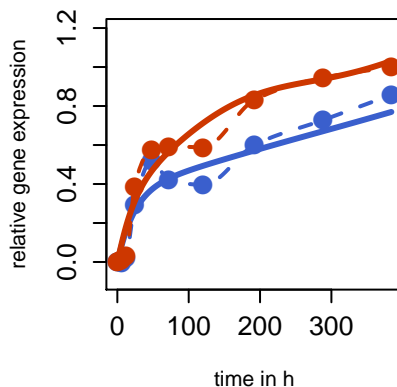**MSX1**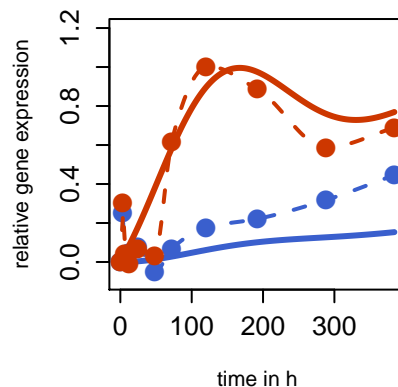**COL2A1**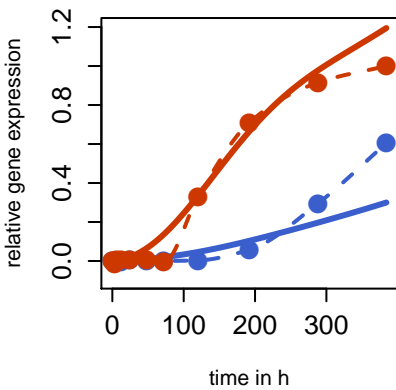**SATB2**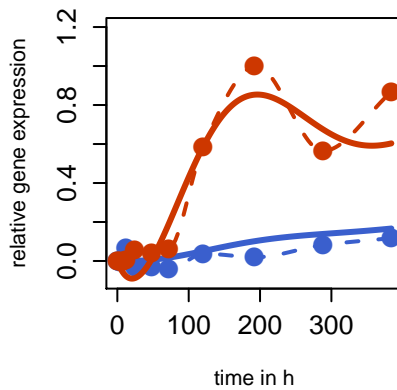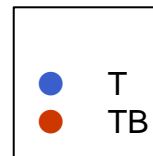

Supplement: Additional file 4 — Figure: Chondrogenesis system, time courses. Comparison of the chondrogenesis system time courses. Each panel displays the results of one gene: the simulated time course (solid line), interpolated measurements (dashed line) and the measured time series (dots) for both data sets (“T” and “TB”). [file 1752-0509-7-1-S4.pdf]
